# Autophagy modulates the effects of bis-anthracycline WP631 on p53-deficient prostate cancer cells

**DOI:** 10.1111/jcmm.12402

**Published:** 2015-02-16

**Authors:** Sylvia Mansilla, Carolina Vizcaíno, Maria A Rodríguez-Sánchez, Waldemar Priebe, José Portugal

**Affiliations:** a Instituto de Biología Molecular de Barcelona, CSIC, Parc Científic de BarcelonaBarcelona, Spain; b The University of Texas MD Anderson Cancer CenterHouston, TX, USA

**Keywords:** autophagy, cell death, PC-3 cells, Sp1, WP631

## Abstract

Treatment of p53-deficient PC-3 human prostate carcinoma cells with nanomolar concentrations of bis-anthracycline WP631 induced changes in gene expression, which resulted in G2/M cell cycle arrest, autophagy and cell death. The presence of 2-deoxy-D-glucose (2-DG), which induces metabolic stress and autophagy, enhanced the antiproliferative effects of WP631. Changes induced by WP631, 2-DG, or co-treatments with both compounds, in the expression of a variety of genes involved in autophagy and apoptosis were quantified by real-time PCR. They were consistent with a raise in autophagy followed by cell death. Some cells dying from G2/M phase showed features of necrosis like early changes in membrane permeability, while others were dying by apoptosis that occurred in presence of little caspase-3 activity. Our results indicate that WP631 is not only an antiproliferative agent acting on gene transcription, but it can also induce autophagy regardless of the presence of other pro-autophagy stimuli. The development of autophagy seemed to improve the cytotoxicity of WP631 in PC-3 cells. Our results indicate that autophagy would enhance the activity of DNA-binding drugs like WP631 that are potent inhibitors of gene transcription.

## Introduction

Diverse responses can be activated in cancer cells during chemotherapy, which depend on the antitumour agent, the dose used and the genetic background 1. Because the loss of the ability of tumour cells to activate apoptosis following treatment with radiation or chemotherapy is one of the most frequent alterations that occur in solid tumours, the elucidation of the pathways that control p53-dependent and p53-independent cell death should provide new strategies for the development of more effective drugs. In this context, it is worth exploring whether bis-anthracycline WP631, which is known to inhibit the binding of Sp1 transcription factor to promoters 2,3 producing extensive changes in cell's transcriptome 4, can undertake its activity in autophagic cells. Autophagy is a cellular process that plays a central role in the integrated stress response 5. It is activated by a variety of stress stimuli, including nutrient starvation, hypoxia or DNA damage, thus it can exert a pro-survival role in cell homoeostasis 6,7. To some extent, this is accomplished through the expression of genes related to autophagy, and thus the situation can be challenged by the treatment with some molecules like bis-anthracycline WP631 that abrogate gene expression.

Autophagy participates in the control of cellular damage in response to stress, limiting tumour growth 6. However, the process of stress survival afforded by autophagy may be a major obstacle to achieving successful chemotherapeutic treatment of cancer. In fact, autophagy appears to play dual roles in cancer by clearing away damaged proteins and organelles, and by preventing genome damage by maintaining energy homoeostasis 8–11. Hence, disruption of autophagy, which reduces cellular fitness, would promote tumourigenesis. Nonetheless, whether autophagy may contribute to chemoresistance or it represents a mechanism of cell death after chemotherapy is still a controversial subject 1,12. To develop successful autophagy-modulating strategies against cancer, we need to better understand how the roles played by autophagy differ depending on the cell type and genetic factors. It is also necessary to determine how autophagic pathways are activated or inhibited by antitumour agents. For clinical use, it is relevant to determine whether enhanced autophagy is a sign of drug responsiveness or resistance 7,13,14. Besides, when a decrease in the apoptotic response occurs during therapy, this may be compensated by alternative cell death mechanisms, including necrosis/necroptosis, mitotic catastrophe, and by the presence of autophagy or senescence 1,15,16.

Bis-anthracycline WP631 bis-intercalates into C/G-rich DNA regions with higher sequence-selectivity and affinity than monomeric anthracyclines 17,18. WP631 is a potent inhibitor of transcription, fundamentally through direct competition with the Sp1 transcription factor for their consensus binding sites in gene promoters both *in vitro* and *in vivo* 2–4,19. In general, nanomolar concentrations of WP631 have a superior cytotoxic activity than monomeric anthracyclines in human cancer cell lines, and they can induce both p53-dependent and p53-independent cell death 20,21. In some human breast carcinoma cells, this occurs through mitotic catastrophe by caspase-dependent and caspase-independent mechanisms 21.

The synthetic glucose analogue 2-deoxy-D-glucose (2-DG) inhibits glycolysis and interferes with protein folding. It can specifically amplify metabolic stress, and its use combined with chemotherapeutic agents has been suggested to increase therapeutic efficacy 22. Furthermore, 2-DG can be used to produce/enhance autophagy in cells 8, providing us with a tool to study the effects of antitumour drugs in cells undergoing autophagy. Autophagy involves the formation of autophagosomes, which are double-membrane structures that enclose and isolate targeted cellular components to travel to the lysosomes, where they are degraded by acidic hydrolases 13. Several genes that regulate the formation of autophagosomes and some of them, such as *LC3* and *Beclin 1*, can be used as molecular markers of autophagy 23, while in turn their expression can be targeted by WP631.

Pharmacological inhibition of autophagy sensitizes cancer cells to chemotherapy, suggesting that suppression of the autophagic pathways is a strategy for cancer treatment 6,24,25. Nevertheless, efficient stimulation of autophagy may also provide a therapeutic strategy for treating resistant cancer cells that show poor apoptotic response 6.

In this paper, we evaluate whether the antitumour activity of WP631 on the p53-defective PC-3 human prostate carcinoma cells is modulated by the autophagy-inductor 2-DG. Nanomolar concentrations of WP631 inhibited cell growth and induced autophagy in PC-3 cells. Given that the induction of autophagy appeared to contribute to chemosensibilization in PC-3 cells, we hypothesize that enhancing autophagy in p53-defective cells can be used as a strategy to improve the sensitivity of tumour cells to DNA-binding agents that inhibit transcription.

## Materials and methods

### Materials and cell culture

Bis-anthracycline WP631 was synthesized as described elsewhere 17. Stocks were prepared as 500 μM solutions in sterile 150 mM NaCl, maintained at −20°C, and brought to the final concentration before use with cell culture medium. 2-deoxy-D-glucose (2-DG), acridine orange, Trypan blue, bafilomycin A1 (BafA1) and 3-(4,5-dimethylthiazol-2-yl)-2,5-diphenyl tetrazolium bromide (MTT) were purchased from Sigma-Aldrich (St. Louis, MO, USA). BrdU (5′-bromo-2′-deoxy-uridine) was from Roche Diagnostics (Madrid, Spain). All other chemicals were reagent or molecular grade, as appropriate.

PC-3 human prostate carcinoma cells were maintained in RPMI-1640 medium (Life Technologies, Prat de Llobregat, Spain) supplemented with 2 mM sodium pyruvate (Life Technologies), 10% foetal bovine serum (Life Technologies), 100 U/ml penicillin, and 100 μg/ml streptomycin, at 37°C in a humidified atmosphere with 5% CO_2_. Exponentially growing cells subculturated at a density of 2.5 × 10^4^ cells/ml were treated with WP631, 2-DG or 2-DG plus WP631 at 37°C at the concentrations specified in Results for different periods of time.

### Cytotoxicity assays

The effect of WP631 on PC-3 cell growth was determined by the MTT-method. Cells subcultured at a density of 2.5 × 10^4^ cells/ml in 96-well microtiter plates (Corning Costar, Corning, NY, USA) in a total volume of 100 μl were incubated with various concentrations of WP631 at 37°C for 72 or 96 hrs. After incubation, MTT was added to each culture, and the dark-coloured crystals produced by viable cells were solubilized. Absorbance was determined at 570 nm by using a BioTek ELx800 microplate reader (BioTek Instruments, Winooski, VT, USA). Moreover, viable cell number was determined based on the exclusion of Trypan blue dye and a haemocytometer.

Apoptosis was quantified and distinguished from necrosis by using the Annexin-V-Fluos staining kit (Roche Diagnostics) and flow cytometry in a Coulter Epics-XL flow cytometer (Beckman Coulter, Hialeah, FL, USA).

### Quantification of acidic vesicular organelles

PC-3 cells at a density of 2.5 × 10^4^ cells/ml were growth in 25 cm^2^ flasks (Corning), and incubated with different concentrations of 2-DG for 24, 48 or 72 hrs. Cells were resuspended in PBS, incubated with 5 μg/ml acridine orange (Sigma-Aldrich) for 15 min. at room temperature, washed twice with PBS, and analysed in a Gallios flow cytometer (Beckman Coulter, Miami, FL, USA). Fluorescence intensity values were used to calculate the relative fluorescence ratio (treated *versus* control cells).

### Analysis of cell cycle distribution by flow cytometry

PC-3 untreated (control) cells and cells treated with either 2-DG, WP631, or co-treated with 2-DG plus WP631 for different times were collected, fixed with 70% ethanol, stained with PI (Propidium iodide; Sigma-Aldrich), and the cell cycle distribution was determined by analysing the nuclei in a Coulter Epics-XL flow cytometer.

### Determination of DNA synthesis and quantification of the mitotic index

DNA synthesis was determined by measuring the incorporation of BrdU by using a fluorescence-conjugated antibody against BrdU (BD Biosciences, San Agustin de Guadalix, Spain), co-stained with PI, and analysed in a Coulter Epics-XL flow cytometer.

To analyse the mitotic fraction, fixed cells were incubated with the anti-phospho-Histone H3 (ser 10) antibody (Merck Millipore, Madrid, Spain) followed by Cy2-conjugated secondary antibody (Jackson ImmunoResearch, Newmarket, UK). Stained cells were then counterstained with PI and analysed for Cy2 and PI fluorescence in a Coulter Epics-XL flow Cytometer.

### Measurements of caspase-3 activity

A bivariate flow cytometry analysis of intracellular caspase-3 activation and apoptotic cell death was used to distinguish between cells dying by apoptosis through activation of caspase-3 from those dying through different routes. Caspase-3 activity assay was performed by incubating cells with PhiPhiLux G1D2 substrate solution (Calbiochem, Merck, Darmstadt, Germany) for 1 hr at 37°C in 5% CO_2_, while apoptosis was assessed by co-staining with Annexin-V-Fluos (Roche Diagnostics). The different samples were immediately analysed in a BD FACSAria flow cytometer (Becton Dickinson, Franklin Lakes, NJ, USA) by using excitations at 488 and 532 nm.

### RNA extraction and quantitative real-time PCR analysis

Total RNA was extracted from control (untreated) cells and from cells treated with 2-DG, WP631 or 2-DG plus WP631, at the concentrations indicated below, for 24 hrs. The UltraspecRNA isolation reagent (Biotecx, Houston, TX, USA) was used following the procedure provided by the supplier. RNA was digested with RNAse-free DNAse I (Roche Diagnostics) in the presence of RNAse inhibitors (RNasin; Promega Biothech Iberica, Madrid, Spain), phenol extracted and precipitated, and the pellet was dissolved in RNAse-free water. The yield and purity of total RNA were assessed spectrophotometrically and RNA integrity examined in an Agilent 2100 Bioanalyzer (Agilent Technologies, Wilmington, DE, USA).

Quantitative real-time PCR (qRT-PCR) experiments were designed and performed in accordance with the MIQE guidelines.^26^ cDNAs were synthesized from 2 μg of isolated RNA obtained from two biological replicates, in a 20 μl reaction volume by using the Transcriptor First Strand cDNA synthesis kit (Roche Diagnostics) following manufacturer's instructions. A set of 10 human genes involved in the response to cellular stress, autophagy and apoptosis, as well as the housekeeping gene *GAPDH*, were selected to be analysed by qRT-PCR (see Results). Gene-specific primers sets were designed using the Primer Express software (Applied Biosystems, Life Technologies, Carlsbad, CA). The primers used for qRT-PCR are listed in Table1. Reactions were performed in triplicate by using the SYBR-Green PCR Master Mix (Roche Diagnostics). Amplification and detection were performed in triplicate in a Roche LightCycler 480 system. PCR conditions included an initial denaturation step at 95°C for 10 min., followed by 45 cycles of a denaturation step at 95°C for 10 sec., an annealing step at 60°C for 30 sec., and an extension step at 72°C for 10 sec. Series of 10-fold dilutions of cDNA were used to generate the standard curves to calculate the efficiency of the amplifications. Reactions in absence of template RNA and in the absence of enzyme were also performed for each primer pair as negative controls. A final dissociation curve was generated to verify that a single product was amplified. Relative expression values of the different genes were calculated from the threshold cycle (Ct) following the ΔΔCt method 27, by using *GAPDH* as internal housekeeping control.

**Table 1:** Primers used for qRT-PCR

Gene*	Primers
*SP1*	For: 5′-CAGCTTCAGGCTGTTCCAAACT-3′
	Rev: 5′-CTGCCAACTGACCTGTCCATT-3′
*MYC*	For: 5′-GGGATCGCGCTGAGTATAAAA-3′
	Rev: 5′-CGAGTTAGATAAAGCCCCGA-3′
*Beclin1*	For: 5′-TCCACAGAAAGTGCCAACAGC-3′
	Rev: 5′-TGTCAAAAAGGTCCCCAGTGA-3′
*ATG3*	For: 5′-ACCACTGTCCAACATGGCAA-3′
	Rev: 5′-GCACGGCACATTTTTGGTTAC-3′
*ATG4B*	For: 5′-ATGATCTTTGCCCAAGCCCT-3′
	Rev: 5′-CCTCCAATCTCGGCCTAGGT-3′
*CASP3*	For: 5′-GCATACTCCACAGCACCTGGT-3′
	Rev: 5′-GAGCCATCCTTTGAATTTCGC-3′
*NFKB1*	For: 5′-GCAGCTCTTCTCAAAGCAGCA-3′
	Rev: 5′-GCTCAAAGTTCTCCACCAGGG-3′
*LC3*	For: 5′-ACCAGCACCCCAGCAAAAT-3′
	Rev: 5′-GCTTCTCACCCTTGTAGCGCT-3′
*BCL2*	For: 5′-AATTTCCTGCATCTCATGCCA-3′
	Rev: 5′-TCACGCGGAACACTTGATTCT-3′
*BAX*	For: 5′-GTCTTTTTCCGAGTGGCAGC-3′
	Rev: 5′-CCAGTTGAAGTTGCCGTCAGA-3′
*GAPDH*	For: 5′-TCTGCCCCCTCTGCTGAT-3′
	Rev: 5′-TTCTCATGGTTCACACCCATG-3′

* The *GAPDH* housekeeping gene was used for data normalization.

### Western blot

Protein was extracted from control and treated PC-3 cells by using a lysis buffer consisting of 50 mM Tris-HCl (pH 8.0), 150 mM NaCl, 5 mM EDTA, 0.5% Igepal (NP-40) and 0.1 mM phenylmethylsulfonyl fluoride, containing 2 μg/ml aprotinin (Sigma-Aldrich) and 1 μg/ml leupeptin (Sigma-Aldrich). Total protein was quantified by the Bradford assay (Bio-Rad, Hercules, CA, USA). About 50 μg of denatured protein was subjected to electrophoresis on SDS-polyacrylamide gels, blotted onto Optitran BA-S85 membranes (Schleicher & Schuell, Dassel, Germany), probed with the specific antibodies for LC3 (MBL, BioNova, Madrid, Spain), Beclin 1 (AbDSerotec; BioNova), Anti-p62/SQSTM1 (Sigma-Aldrich), Anti-PARP (Roche Diagnostics) and β-tubulin (Merck Millipore), incubated with secondary antibodies (Jackson ImmunoResearch) and detected by using Luminol (Sigma-Aldrich).

### Statistical analysis

Statistical analysis was performed with SPSS v.21 (IBM Corp., Armonk, NY, USA). Results represent the mean ± SD, or mean ± SEM values, from three independent experiments. Statistical differences in gene expression between control, untreated cells, and each of the treatments were evaluated by the unpaired Student's *t*-test. One-way (anova) and further Tukey's post-hoc test were performed for multiple comparisons of differences in the relative gene expression after the various treatments.

## Results

### Autophagy potentiates the antiproliferative effects of WP631 on PC-3 human prostate carcinoma cells

**Fig 1:**
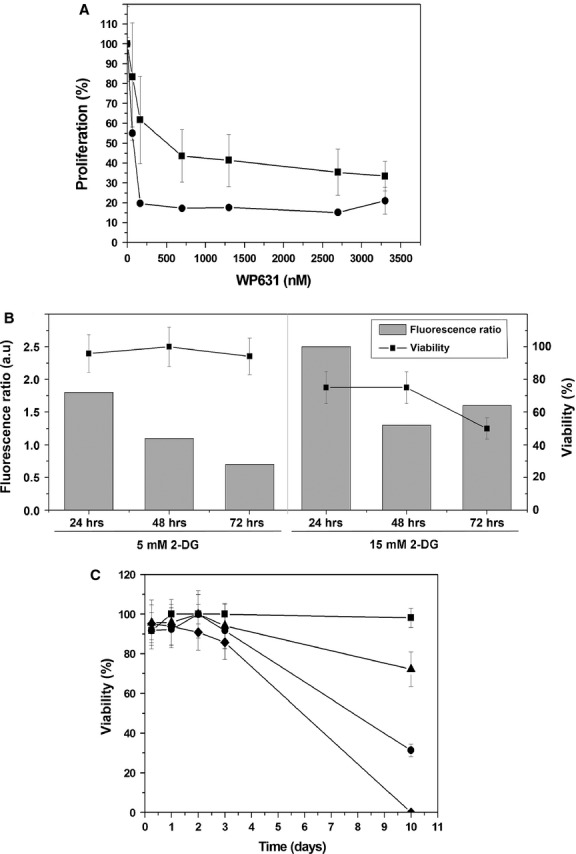
Effects of 2-DG and WP631 on PC-3 human prostate carcinoma cells. (A) Proliferation of PC-3 cells exposed to a range of concentrations of WP631 for 72 hrs (▪) or 96 hrs (•). Data are mean ± SEM from six independent experiments. (B) Accumulation of acridine orange (columns) and cell viability (lines) in PC-3 cells treated with 5 or 15 mM 2-DG for 24, 48 and 72 hrs, respectively. Data are expressed as relative acridine orange fluorescence ratio (treated/control cells), and the percentage of cells excluding Trypan blue dye (mean ± SD from three independent experiments). (C) Viability of PC-3 cells, measured by Trypan blue exclusion, after treatment with 5 mM 2-DG, 165 nM WP631, or co-treatment 5 mM 2-DG plus 165 nM WP631. Untreated control cells (▪), cells treated with 2-DG (▴), WP631 (•) or 2-DG plus WP631 (♦) respectively. Data are expressed as a percentage of viable cells (mean ± SD from three independent experiments).

As the first step towards characterizing the effects of WP631 on PC-3 human prostate carcinoma cells, we evaluated its antiproliferative effects by the MTT-assay, by using a wide range of drug concentrations for 72 and 96 hrs (Fig.1A). Low nanomolar concentrations of WP631 strongly inhibited cell growth, although the drug concentration required to inhibit the growth of PC-3 cells by 75% (its IC_75_) was only quantifiable after 96-hrs treatment (IC_75_ = 165.0 ± 0.8 nM), see Figure1A.

The glucose analogue 2-deoxy-D-glucose (2-DG) was used to examine whether activating autophagy in PC-3 cells may alter the cytotoxic effects of WP631. To define optimal experimental conditions, PC-3 cells were treated with either 5 mM or 15 mM 2-DG for 24, 48, and 72 hrs respectively, stained with acridine orange and analysed by flow cytometry (Fig.1B), taking advantage of the acridine orange accumulation on acidic organelles (autophagosomes and autolysosomes) 25. In parallel, we determined cell viability after these treatments by Trypan blue staining (Fig.1B). The ratios between the mean acridine orange fluorescence in cells treated with either 5 or 15 mM 2-DG and in untreated cells are shown in Figure1B, together with the corresponding percentages of cell viability. The presence of 2-DG increased the fluorescence ratios in a dose-dependent way, which implies that acidic vesicular organelles were formed, consistent with the induction of autophagy. Altogether, the experiments shown in Figure1 allowed us to determine the concentration of 2-DG inducing autophagy without massive cell death that was suitable for combination studies. The highest fluorescence intensity was obtained in cells treated with 15 mM 2-DG, but this treatment induced high mortality [50% of the treated cells were positive for Trypan blue staining (Fig.1B)]. Hence, a concentration of 5 mM 2-DG was selected for further experiments to investigate the effect of double treatments for longer periods.

Quantification of the number of viable cells in the presence of either 5 mM 2-DG or 165 nM WP631 showed that both compounds had similar effects in cell viability after 5-hrs or 3-day treatments (Fig.1C). However, WP631 was more cytotoxic than 2-DG, as measured by the ability of living cells to exclude Trypan blue after continuous treatments. About 69% cells treated with 165 nM WP631 for 10 days were dead and detached, floating, in the culture medium, while 2-DG only produced 28% mortality (Fig.1C). The cytotoxic effect of WP631 was strongly enhanced by co-treatment with 2-DG since almost 100% cells died after 10-day continuous treatment, while most of the untreated cells remained alive (Fig.1C).

### WP631 induces autophagy in PC-3 cells, which is followed by transient cell cycle arrest in G2/M phase

**Fig 2:**
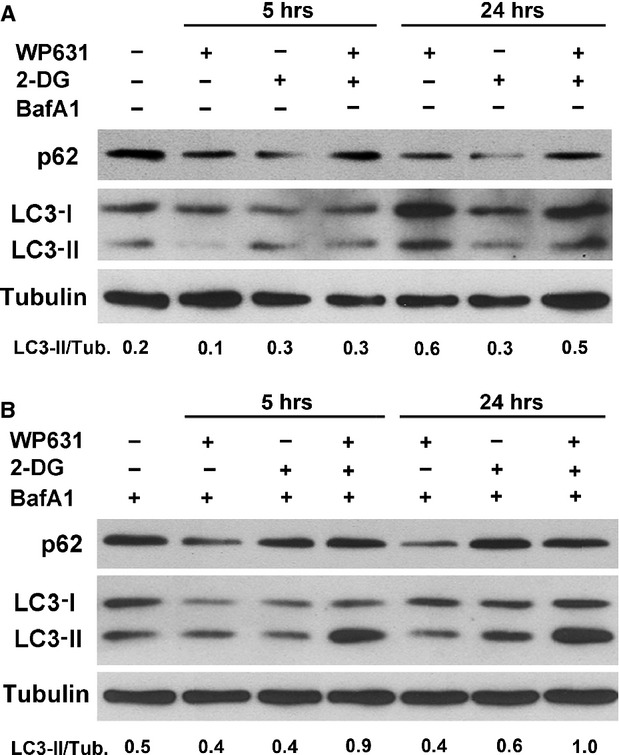
Western blot analyses of the time-dependent effects of 2-DG, WP631 or co-treated with 2-DG plus WP631 on p62/SQSTM1, LC3-I and LC3-II levels in PC-3 cells. (A) Immunoblots of total protein extracted from untreated cells, and from cells treated with 5 mM 2-DG, 165 nM WP631 or co-treated with 5 mM 2-DG plus 165 nM WP631 for 5 and 24 hrs, in which tubulin blot represents a protein loading control. The LC3-II/tubulin ratio was calculated to compare the normalized amount of LC3-II among samples as indicator of autophagy. Protein p62/SQsSTM1 degradation was also examined to detect autophagic flux. This figure shows a representative experiment of experiments performed in duplicate with similar results. (B) Immunoblots of total protein extracted from untreated cells, and from cells treated with 5 mM 2-DG, 165 nM WP631 or co-treated with 2-DG plus WP631 in the presence of 100 nM bafilomycin A1 (Baf A1), which blocks the fusion between autophagosomes and lysosomes, for 5 and 24 hrs. Other details as in panel A.

Western blot analyses revealed the rise in the levels of the autophagy marker LC3-II in PC-3 cells treated with 5 mM 2-DG for 24 hrs (Fig.2), consistent with the acridine orange staining experiments described above. We used time-course immunoblotting analyses to compare the differences in the levels of LC3-II between cells treated with WP631 or co-treated with 2-DG plus WP631, and compare them to those induced by 2-DG alone. Treatments with 165 nM WP631 raised the levels of the lipidated LC3-II form in keeping with autophagy activation. Co-treatment with 2-DG plus WP631 for 24 hrs produced higher accumulation of the LC3-II marker (Fig.2A). Because LC3-II may be itself degraded during autophagy 28, we sought further evidence on whether WP631, either alone or in presence of 2-DG, activated autophagy by examining the autophagic flux in the presence of 100 nM BafA1 that blocked the fusion between autophagosomes and lysosomes (Fig.2B). WP631 increased the LC3-II levels in 24-hrs treatments, while co-treatments with 2-DG plus WP631 caused the highest LC3-II levels. Moreover, p62/SQSTM1 degradation was examined after the different treatments both in the absence (Fig.2A) and in the presence of BafA1 (Fig.2B). 2-DG alone produced clear p62/SQSTM1 degradation. This degradation was lower when BafA1 was present as it blocked the fusion between autophagosomes and lysosomes (Fig.2B). Nevertheless, a decline in p62 levels was observed upon treatments with WP631. Given that p62 function is not limited solely to autophagy, we consider it could tentatively indicate a stress response of drug-treated cells that resulted in its partial degradation. Altogether, our results denoted that autophagy was involved in an early response of PC-3 cells to WP631, and that the combination of 2-DG plus WP631 attained the highest levels of autophagy.

**Fig 3:**
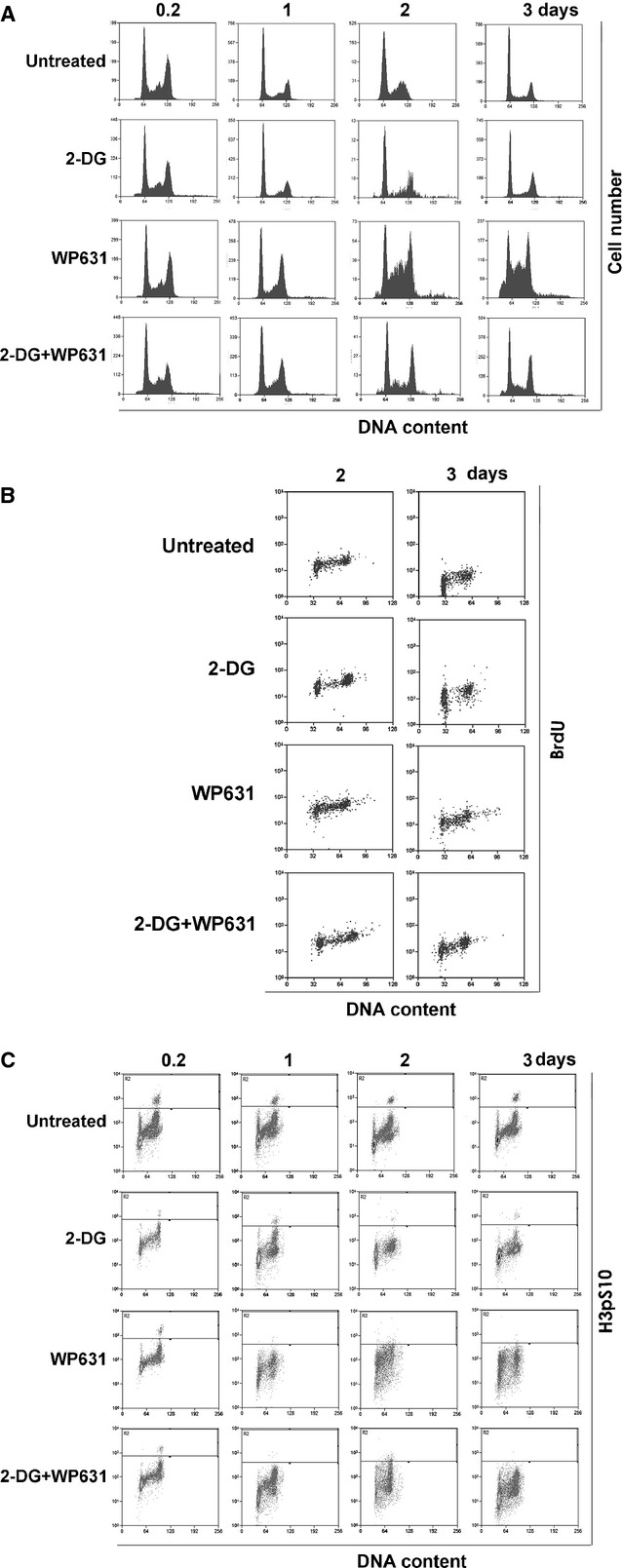
Cell cycle distribution of PC-3 cells treated with 2-DG, WP631, or co-treated with 2-DG plus WP631. (A) Flow cytometry analysis of the time-dependent changes in cell cycle distribution of PC-3 cells treated with 5 mM 2-DG, 165 nM WP631, or 5 mM 2-DG plus165 nM WP631. Both adherent (attached) and detached (floating) cell populations were analysed and their distribution in the different phases of the cell cycle quantified (see Table2). The figure shows a representative experiment of experiments performed in duplicate with similar results. (B) Time-course analysis of DNA synthesis in PC-3 cells treated with 165 nM WP631 or 5 mM 2-DG plus 165 nM WP631. DNA synthesis (BrdU incorporation) was measured by flow cytometry after 48- or 72-hrs treatments. (C) Quantification of the mitotic index by bivariate staining with PI and an antibody against the specific mitotic marker H3pS10.

Changes in cell-cycle distribution after treatments with 2-DG, WP631, or the combined treatment with 2-DG plus WP631, were time-dependent (Fig.3A). Both adherent and detached cells were pooled together for cytometric analysis. A quantification of the percentage of cells found in the different phases of the cell cycle, detected by flow cytometry, is shown in Table2. Both untreated cells and those treated with 2-DG accumulated in G1 phase, yet the percentage of cells decreased upon treatment. On the other hand, cells treated with WP631 alone, or co-treated with 2-DG, mainly accumulated in G2/M phase (Fig.3A and Table2). After halting in G2/M phase, cells accumulated in what, at first glance, could be considered an S phase peak. However, we observed that those cells were not synthesizing DNA, as they did not incorporate BrdU (Fig.3B). These results indicated that the cytometric S-like peak corresponded to cells dying from G2/M rather than to cells accumulating in S phase (seemingly, cells dying from G2/M phase underwent DNA degradation and registered as S-like phase). Moreover, the presence of the mitotic Histone H3pS10 variant (ser-10-phosphorylated Histone H3) was evaluated in cells treated either with WP631 or with 2-DG plus WP631 to distinguish whether cells arrested in G2/M where in G2 or entering mitosis before they were committed to dying. Treated cells did not show any increase in their mitotic index compared to untreated cells (Fig.3C), thus they were not in mitosis. It could still be the case that some cells entered a faulty mitosis and, after a swift slippage, they became a fraction of the S-like peak.

**Table 2:** Time-dependent changes in cell cycle distribution (%) of PC-3 cells treated with 2-DG, WP631 or 2-DG plus WP631

Treatment	Phase	Time (days)			
		0.2	1	2	3
Untreated	SubG1	0.79	0.10	1.01	0.56
	G1	38.69	52.54	54.01	52.77
	S	23.48	17.50	18.97	21.37
	G2/M	36.57	29.72	27.33	25.98
5 mM 2-DG	SubG1	1.65	0.33	2.62	0.93
	G1	39.95	57.65	46.45	46.34
	S	24.97	14.18	19.4	18.14
	G2/M	32.73	28.1	34.32	35.19
165 nM WP631	SubG1	3.27	0.26	2.33	6.82
	G1	41.66	37.40	25.59	27.92
	S	29.55	18.42	35.16	32.87
	G2/M	25.07	44.70	34.78	30.69
5 mM 2-DG + 165 nM WP631	SubG1	1.76	0.77	2.40	3.56
	G1	44.51	42.72	35.71	37.19
	S	32.92	20.66	21.95	19.53
	G2/M	19.92	36.56	39.10	38.83

### WP631 changes the expression of genes involved in autophagy and apoptosis

Given that WP631 is a potent agent acting on transcription (see Introduction), we sought to disclose whether the rising of autophagy in PC-3 cells may correlate with changes in gene expression, and if those changes affected the expression of genes involved in apoptosis. To this end, we selected a set of ten genes involved in the control of apoptosis, autophagy or/and they are known targets of WP631, namely: *Sp1*, *c-Myc*, *Beclin 1*, *ATG3*, *BCL2* (*Bcl-2*), *LC3*, *ATG4B*, *BAX*, *CASP3* (*caspase-3*) and *NF-kB1*.

**Fig 4:**
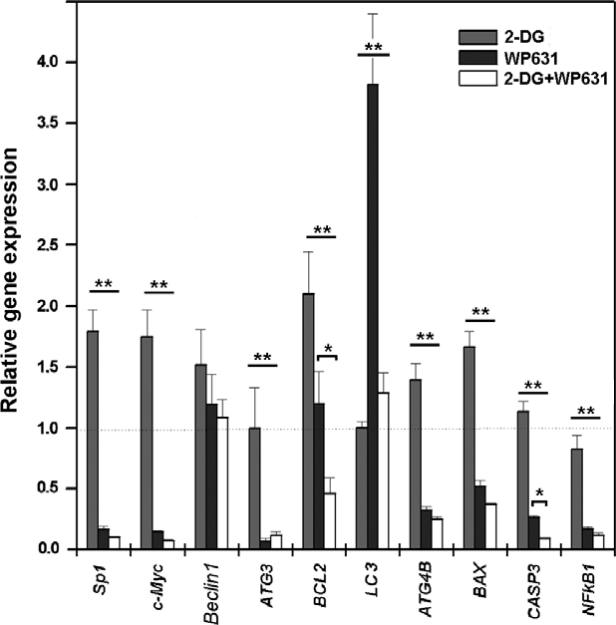
Relative gene expression in PC-3 cells. Changes in gene expression after treatments with 5 mM 2-DG, 165 nM WP631, or co-treatment with 5 mM 2-DG plus 165 nM WP631 for 24 hrs were quantified by qRT-PCR, compared to gene expression in untreated cells. Data are mean ± SD from three independent experiments. For all the genes analysed by qRT-PCR, statistical analyses were performed to evaluate the differences in gene expression. Multiple group comparisons of expression of every gene across different treatments were assessed by anova with Tukey's post-hoc test for two-sample comparisons between treatments (**P < 0.01; *P < 0.05). An unpaired Student's *t*-test that compares changes in gene expression between treated and untreated cells for every treatment is documented in Table3.

Gene expression was quantified by qRT-PCR after 24-hrs treatments. Table3 presents a complete data set for all the genes analysed after treatments with 5 mM 2-DG, 165 nM WP631, or co-treatment with 5 mM 2-DG plus 165 nM WP631. Table3 includes the normalized experimental Ct-values and it reports the statistical pairwise analysis of the differences in gene expression between untreated cells and cells undergoing any of the three treatments. A comparison of the relative expression profiles for the whole set of genes is shown in Figure4. Changes in gene expression depended on the treatment, and both up- and down-regulated genes were observed. In general, differences in the relative gene expression were statistically significant (details are reported in Table3 and legend to Fig.4).

**Table 3:** Relative expression of genes analysed by qRT-PCR in PC-3 prostate carcinoma cells after treatment with 2dG. WP631 or 2dG plus WP631. Housekeeping *GAPDH* gene was used for data normalization

Gene	Accesion no.	Average normalized ΔCt				Fold regulation			Up/down-regulation			*P*-value*		
		Control	2dG	WP631	2dG+WP631	2dG	WP631	2dG+WP631	2dG	WP631	2dG+WP631	2dG	WP631	2dG+WP631
*Sp1*	NM_138473	−2.8	−1.9	−5.3	−6.0	1.8	0.2	0.1	1.8	−6.0	−9.8	6.3E-04	1.1E-04	8.2E-05
*c-Myc*	NM_002467	−3.3	−2.5	−6.0	−7.0	1.8	0.2	0.1	1.8	−6.8	−13.5	2.0E-03	9.3E-05	6.6E-05
*Beclin1*	NM_003766.3	−2.0	−1.4	−1.7	−1.8	1.5	1.2	1.1	1.5	1.2	1.1	1.4E-02	1.8E-01	5.4E-01
*ATG3*	NM_022488.3	−0.2	−0.3	−4.1	−3.3	1.0	0.1	0.1	1.0	−14.7	−8.4	7.2E-01	5.5E-03	6.9E-03
*BCL2*	NM_000633.2	−7.4	−6.3	−7.3	−8.6	2.1	1.2	0.5	2.1	1.1	−2.2	7.2E-04	7.0E-01	5.0E-03
*LC3*	NM_032514.3	−5.6	−5.6	−3.9	−5.3	1.0	3.8	1.3	1.0	3.8	1.3	9.0E-01	1.8E-02	3.9E-02
*ATG4B*	NM_013325.4	−1.8	−1.3	−3.4	−3.8	1.4	0.3	0.3	1.4	−3.1	−4.0	2.5E-03	4.0E-06	3.0E-06
*BAX*	NM_004324.3	−1.1	−0.3	−2.0	−2.5	1.7	0.5	0.4	1.7	−1.9	−2.7	1.4E-03	3.4E-04	8.5E-05
*CASP3*	NM_004346.3	−2.4	−2.3	−4.4	−5.9	1.1	0.3	0.1	1.1	−3.7	−10.9	2.2E-02	1.0E-06	1.0E-06
*NFkB1*	NM_001165412.1	−1.7	−2.0	−4.3	−4.8	0.8	0.2	0.1	−1.2	−5.9	−8.3	2.9E-02	5.7E-05	4.6E-05
*GAPDH*	NM_002046	0.0	0.0	0.0	0.0	1.0	1.0	1.0	1.0	1.0	1.0	0.0E+00	0.0E+00	0.0E+00

* Unpaired Student's *t*-test (control *versus* treated cells).

While WP631 mainly induced gene repression, treatment of PC-3 cells with 5 mM 2-DG augmented significantly the expression of seven genes, and only *NF-kB1* was down-regulated (Fig.4 and Table3). The glucose analogue 2-DG up-regulated the expression of the early-response genes *Sp1* and *c-Myc*, and the inductor of autophagy *Beclin 1*, which suggests that those changes in gene expression were a response to metabolic/cellular stress induced by 2-DG. Indeed, *Beclin 1* was up-regulated by every treatment, although the differences in the degree of up-regulation induced by any treatment were almost indistinguishable from a mere stochastic effect. Therefore, we used Western blot to confirm the concomitant enhancement of Beclin 1 protein levels upon treatments (see below).

WP631 changed the expression of eight genes significantly (Table3 and Fig.4), of which seven were down-regulated. *Beclin 1*, which is a key player in the autophagic response, was up-regulated by WP631 (1.2-fold, *P* = 0.18). Changes in the expression of the anti-apoptotic *Bcl-2* and the pro-apoptotic *BAX* were observed in the different treatments (Table3 and Fig.4). *Bcl-2* was significantly down-regulated in co-treatment experiments (*P* < 0.01), while either 2-DG (*P* < 0.01) or WP631 (*P* = 0.7) enhanced its expression, while *BAX* was down-regulated by both WP631 and the co-treatments.

Combined treatment with 5 mM 2-DG plus 165 nM WP631 inhibited the expression of eight genes significantly (Table3 and Fig.4). As in treatments with WP631 alone, *LC3* expression was up-regulated (*P* < 0.05). Unlike in PC-3 cells treated with 2-DG, in which *Sp1* and *c-Myc* were up-regulated, the treatments with WP631 alone, or in its combination with 2-DG, resulted in a strong inhibition of the Sp1-dependent genes *c-Myc and Sp1*, in agreement with the great effects of WP631 on gene transcription 4,20. This occurred together with the reduced transcription by WP631 of the autophagy-related genes *ATG3* and *ATG4B*, which are involved in the processing of LC3 and the autophagosome biogenesis 29. The down-regulation of those genes that participate in LC3 lipidation did not appear to affect the rise in the LC3-II levels after 24-hrs treatments (Fig.2), thus indicating that the remaining levels of transcription were enough for autophagy to occur, or that other pathways could be involved in the formation of LC3-II upon treatments with WP631. *NF-kB1* expression was down-regulated by every treatment, which suggest that both WP631 and 2-DG can abrogate the pro-survival *NF-kB* pathway. The expression of *CASP3* (*caspase-3*) was slightly up-regulated by 2-DG. An intriguing aspect of the qRT-PCR results was the down-regulation of *CASP3* by the other treatments, with significant superior repression by the co-treatments (*P* < 0.05; Fig.4). Given that changes in the expression of caspase-3 may preclude the final fate of PC-3 cells, we performed experiments aimed at understanding the routes that may commit cells to dying and the role played by caspase-3, which are described below. In general, the patterns of gene expression in PC-3 human prostate carcinoma cells treated with WP631 or co-treated with 2-DG plus WP631 were equivalent (see legend to Fig.4), although, as described above, down-regulation of *Bcl-2* was observed after the co-treatments.

### WP631 induces early changes in the permeability of cell membranes and caspase-3 independent apoptosis in PC-3 cells

**Fig 5:**
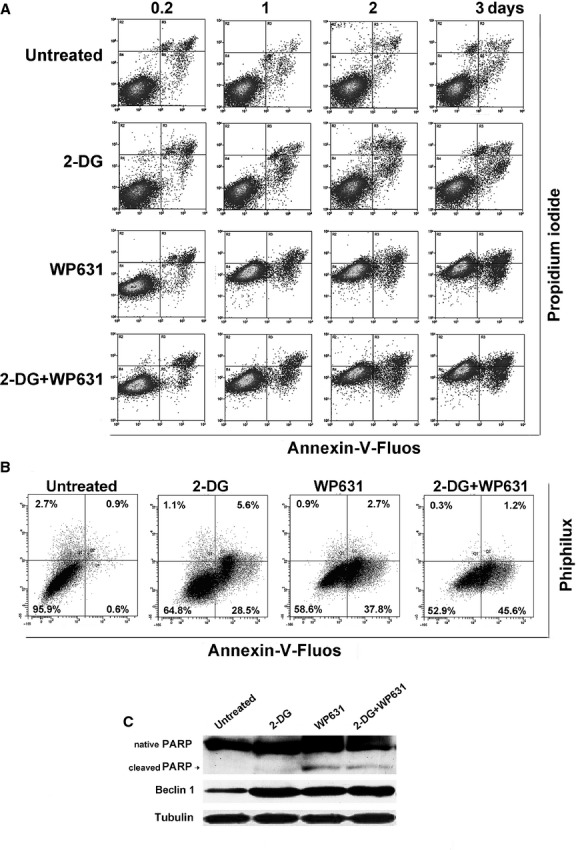
Analysis of cell death in PC-3 human prostate carcinoma cells treated with 2-DG, WP631 or co-treated with 2-DG plus WP631. (A) Time-course bivariate flow cytometric analysis. Adherent (attached) and detached (floating) cell populations were pooled together and stained with Annexin-V-Fluos and PI. A quantification of these plots is summarized in Table4. (B) Simultaneous assessment by flow cytometry of apoptotic cells (Annexin-V-Fluos staining) and caspase-3 activation (cleavage of the fluorescent substrate PhiPhilux G1D2) in cells treated continuously for 3 days. (C) Western blot analysis of the presence of cleaved PARP protein, and of changes in Beclin 1 protein levels in PC-3 cells treated for 3 days. This panel shows a representative experiment of experiments performed in duplicate with similar results.

Double staining with Annexin-V-Fluos and PI was used to determine the fate of cells dying after the different treatments. Based on the staining pattern, cells were classified as: apoptotic (high levels of Annexin-V-Fluos, which detects the presence of cell-surface phosphatidylserine, and low PI staining), necrotic (high levels of PI staining, caused by changes in membrane permeabilization, and low staining of Annexin-V-Fluos), and secondary apoptosis/necrosis (high levels of staining with both Annexin-V-Fluos and PI). Flow cytometry showed that untreated, control, cells were mostly viable throughout the period analysed, while a time-dependent increase of cell death was observed for all the treatments (Fig.5A). PC-3 cells treated with 5 mM 2-DG mainly died through apoptosis. Treatment with 165 nM WP631 produced early small change in membrane permeability in PC-3 cells, characterized by a slight increase in PI staining, which was followed by massive apoptosis (Fig.5A).

Co-treatments with 2-DG plus WP631 induced higher levels of apoptosis (17.9% Annexin-V-Fluos positive cells after 72 hrs) compared to 2-DG (9.1%) or WP631 (15.7%) (Fig.5A and Table4). A quantification of the percentages of necrosis, apoptosis, and secondary apoptotic/necrotic cell death after the different treatments is summarized in Table4. Having established that apoptosis was induced by the different treatments, we examined whether caspase-3 was involved. PC-3 cells treated with 2-DG, WP631 or 2-DG plus WP631 up to 3 days displayed both Annexin-V-Fluos staining and caspase-3 activity (*i.e*. cleavage of the Phiphilux substrate) (Fig.5B). However, the highest enzymatic activity was observed in treatments with 2-DG. The presence of little caspase-3 activity after treatments with WP631 alone was in agreement with the inhibition of its gene expression by the drug (see above).

**Table 4:** Quantification of cell death in PC-3 cells treated with 2-DG, WP631 or 2-DG plus WP631 for different periods of time. Percentages were quantified by double staining with Annexin-V-Fluos and propidium iodide

Treatment	Cell death type	Time (days)			
		0.2	1	2	3
Untreated	Viable	90.72	95.15	94.50	91.31
	Apoptosis	3.82	4	1.93	4.36
	Necrosis	0.22	0.01	0.9	2.26
	Secondary apoptosis/necrosis	5.24	0.85	2.60	2.07
5 mM 2-DG	Viable	90.16	88.83	86.17	84.90
	Apoptosis	3.69	8.31	6.63	9.09
	Necrosis	0.38	0.01	0.97	0.77
	Secondary apoptosis/necrosis	5.77	2.85	6.23	5.24
165 nM WP631	Viable	89.95	83.25	80.54	60.15
	Apoptosis	4.09	8.69	8.16	15.69
	Necrosis	0.05	2.21	4.96	12.84
	Secondary apoptosis/necrosis	5.91	5.85	6.34	11.32
5 mM 2-DG + 165 nM WP631	Viable	88.86	86.07	75.38	64.49
	Apoptosis	4.75	8.93	13.23	17.90
	Necrosis	0.25	0.18	4.06	7.49
	Secondary apoptosis/necrosis	6.15	4.82	7.33	10.1

To gain further insights into the pathways and mechanisms involved in cell death, we also examined the DNA-damage response protein poly [ADP-ribose] polymerase (PARP) by immunoblotting. PARP was examined because it can be involved in the sensitivity of some p53-defficient cells to drug-induced apoptosis 30. Figure5C shows Western blot analyses of PARP cleavage and Beclin 1 levels. A slight cleavage of PARP was observed in the presence of WP631 and 2-DG plus WP631 consistent with emerging apoptotic cell death, whereas it was almost undetectable in treatments with 2-DG, even though this treatment was accompanied by a higher caspase-3 activity (cf. panels C and B in Fig.5). The levels of the autophagy-related Beclin 1 increased after all the treatments, in line with the qRT-PCR results (cf. Figs4 and 5C) and, in turn, with the rise of autophagy (Fig.2).

## Discussion

The experiments described here were designed to answer the question as to whether DNA-binding drugs can be effective inductors of cell death in the presence of high levels of autophagy, because autophagy may play a key role in regulating cell death induced by anticancer agents 1,6,7,11,13. Taking into consideration the complex relationship between cell death and the autophagic pathways, we have explored the potential interactions and problems that may arise when p53-deficient cells are treated with DNA-binding agents while autophagy is taking place. To this end, we have examined PC-3 human prostate carcinoma cells, in which autophagy (augmented by the glucose analogue 2-DG) was active during the treatment with bis-anthracycline WP631, and analysed changes in gene expression and in the cell death routes followed by cells undergoing autophagy.

Co-treatment of PC-3 human prostate carcinoma cells with WP631 plus 2-DG reveals combined antiproliferative effects (Fig.5A) that are in keeping with that conventional chemotherapeutic agents (WP631 is an anthracycline that binds to DNA 17,18) may be used together with pathway-targeted agents (the pro-autophagic 2-DG compound targets glycolysis 8) to reach synergistic anticancer effects. In the context of the therapeutic targeting of the cancer cell metabolism and autophagy 22,31, our results may be used to design new strategies based on strong inhibitors of transcription like WP631. Remarkably, we have observed that WP631 alone can induce some time-dependent autophagy (Fig.2), which seems to enhance its cytotoxicity rather than to disrupt it.

Chiefly, there was no significant difference between the gene expression profiles in cells treated with WP631 or co-treated with WP631 plus 2-DG (Fig.4). It is noteworthy that WP631 was superior to 2-DG in enhancing the expression of the autophagy-related *LC3* gene. Besides, LC3-II, the active form of the *LC3* gene product, was augmented after treatment (Fig.2). The comparison of LC-3- II levels in PC-3 cells upon the different treatments is considered an accurate indicator of autophagy 32. WP631 can compete with Sp1 transcription factor for binding to their consensus sites in gene promoters 2,19, and it circumvents a type of multidrug resistance by both its ‘anti-transcriptional effect' and *via* its direct interaction with the efflux pump 33. While the changes observed in the transcription of several genes (Fig.4) are coherent with direct gene targeting by WP631, it cannot be ruled out we were also observing some effects that are not directly related to WP631 binding to DNA. Indirect effects may arise after the transcriptional up-regulation of several genes (Fig.4 and Table3), owing to the response of cells to general stress generated by the exposure to WP631 and/or 2-DG.

2-DG up-regulates several genes involved in autophagy in PC-3 cells (Fig.4) in agreement with its pro-autophagic role. Moreover, other genes that can be involved in proliferation, like *c-Myc*, were also up-regulated by this compound. Meanwhile, WP631 inhibited *c-Myc* transcription, which is likely to occur by competition with the Sp1 transcription factor for their consensus binding sites in the c-*Myc* promoters. Those changes in gene expression can reflect the response of PC-3 cells to stress. Although c-Myc can be necessary for apoptosis under certain conditions and it can induce apoptosis by p53-independent mechanisms 34, its down-regulation by WP631 indicates that it plays a marginal role in the apoptotic cell death observed under our experimental conditions. The rise of apoptosis in PC-3 cells while autophagy is operative suggests a role for *Bcl-2* and *Beclin 1* in the cellular response. Both genes were up-regulated by 2-DG or WP631, whereas co-treatments reduced *Bcl-2* expression. Although the Bcl-2 family of proteins was initially characterized as cell death regulators, they might also control autophagy 35, in line with our results. Furthermore, Beclin 1 network regulates autophagy, and it can be considered a measure of autophagy competence 6,36. For each treatment, our results speak for the occurrence of a co-regulation of the pathways that bring PC-3 cells to both autophagy and apoptosis. On the other hand, because the *Bcl-2* family regulates 2-DG toxicity 37 the down-regulation of *Bcl-2* by the co-treatments could augment toxicity. The relatively high levels of *Beclin 1* expression and of the protein it encodes (Figs4 and 5C), as well as p62/SQSTM1 degradation, are consistent with the rise of autophagy with LC3 being responsible for recruiting p62 into autophagosomes 38. Nevertheless, p62 degradation also occurred in WP631-treated cells in the presence of autophagic flux inhibitors (Fig.2B). This would indicate that p62 function was not limited solely to autophagy in those cells, but it is associated to other processes such as stress response of drug-treated cells, which could result in its partial degradation. Down-regulation of *NF*κ*B1* after treatments with WP631 (Fig.4) suggests this compound may prevent the role of p62 as activator of NFκB 39.

PC-3 human prostate carcinoma cells treated with WP631 or 2-DG, as well as co-treated ones, were committed to dying (Figs1C and 5A). Changes in gene expression, including those in genes that play key roles in autophagy and apoptosis, failed to prevent cell death. Under some experimental conditions, those changes encompass the down-regulation of the pro-apoptotic *BAX* (Fig.4). Death occurred after the rise of autophagy, but early necrosis and, fundamentally, apoptosis were observed, consistent with that autophagy would constitute a futile attempt to adapt to stress rather than a cell death mechanism 12.

Cells dying from G2/M phase showed some features of necrosis, as early changes in membrane permeability, and ensuing apoptosis occurred in the presence of little caspase-3 activity (Fig.5B). Given the low levels of caspase-3 after the different treatments, it may be argued that other caspases were involved in the apoptotic response. The low induction of caspase-3 by WP631 in PC-3 cells is at variance with the high activation of caspase-3 in an advanced ovarian cell line 40. Those dissimilarities in caspase-3 activation could originate from differences in the genetic background of prostate and ovarian cells, including the status of their *p53* gene. WP631 seems to activate the cleavage of PARP, which is at variance with the concept that PARP inhibition may represent a way of targeting p53-deficient cells 30 as WP631 targets these cells efficiently. The presence of some activation (cleavage) of PARP and the augmented levels of Beclin 1 protein (Fig.5C) could promote caspase-8 degradation 41, thus caspase-8 seems an unlikely candidate to trigger apoptosis.

Autophagy stimulators such as 2-DG induce metabolic stress that can be useful not only for cancer prevention and treatment 8, but also as advantageous modulators of anticancer chemotherapy 7,42. While previous reports have mainly indicated the combined effect of inhibiting apoptosis and autophagy can increase the cytotoxicity of certain compounds 24, our results highlight that enhancing autophagy during treatment with DNA-binding drugs might reach a complementary effect, at least in p53-deficient cells, consistent with that autophagy would become the main death inducing pathway in apoptosis-deficient cells 7. Bis-anthracycline WP631 acts at different pathway levels including autophagy (Figs2 and 4), which makes it an attractive compound. Our studies by using PC-3 cells have potential clinical implications as they suggest that WP631 can be a useful therapeutic agent for treating prostate cancer, providing a rationale for its use in combination with 2-DG. Activation of autophagy may be used to enhance the anticancer effects of WP631 and other potent transcription inhibitors.
